# COVID-19 Impact on Public Health, Environment, Human Psychology, Global Socioeconomy, and Education

**DOI:** 10.1155/2022/5578284

**Published:** 2022-01-11

**Authors:** Youssef Miyah, Mohammed Benjelloun, Sanae Lairini, Anissa Lahrichi

**Affiliations:** ^1^Laboratory of Materials, Processes, Catalysis, and Environment, University Sidi Mohamed Ben Abdellah, School of Technology, Post Office Box 2427, Fez, Morocco; ^2^Laboratory of Biochemistry, Faculty of Medicine and Pharmacy, University Sidi Mohamed Ben Abdellah, Fez, Morocco

## Abstract

The end of the year 2019 was marked by the introduction of a third highly pathogenic coronavirus, after SARS-CoV (2003) and MERS-CoV (2012), in the human population which was officially declared a global pandemic by the World Health Organization (WHO) on March 11, 2020. Indeed, the pandemic of COVID-19 (Coronavirus Disease 19) has evolved at an unprecedented rate: after its emergence in Wuhan, the capital of the province of Hubei of the People's Republic of China, in December 2019, the total number of confirmed cases did not cease growing very quickly in the world. In this manuscript, we have provided an overview of the impact of COVID-19 on health, and we have proposed different nutrients suitable for infected patients to boost their immune systems. On the other hand, we have described the advantages and disadvantages of COVID-19 on the environment including the quality of water, air, waste management, and energy consumption, as well as the impact of this pandemic on human psychology, the educational system, and the global economy. In addition, we have tried to come up with some solutions to counter the negative repercussions of the pandemic.

## 1. Introduction

**Figure 1:**
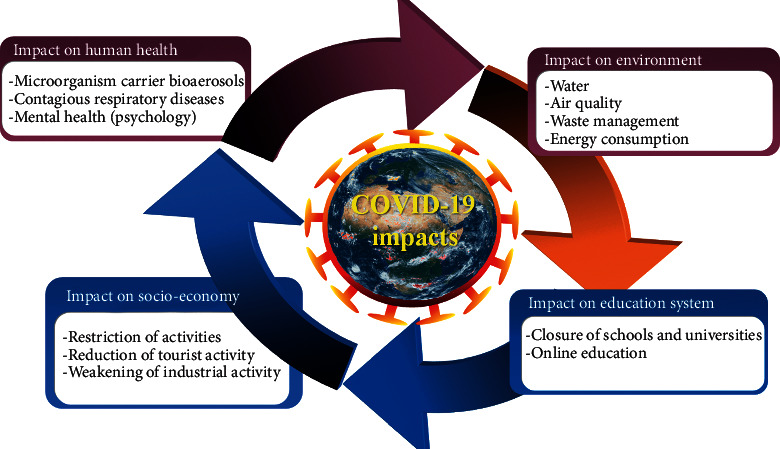
COVID-19 impacts on human health, environment, education system, and socioeconomy.

Today, our world is facing the pandemic of COVID-19 which has not only suspended activity in various key sectors of the economy but has also put the health of the world's population at risk [1, 2]. Coronaviruses are frequent ribonucleic acid viruses, of the Coronaviridae family, which are responsible for digestive and respiratory infections in humans and also in animals [3]. The virus owes its name to the shape of its viral particles, bearing growths that evoke a crown. It is an invisible threat that worries the whole world. COVID-19 first appeared in Wuhan in December 2019 and caused fatal respiratory infections, and then, it spread gradually in the world and, thus, became a global pandemic, triggering a health crisis (WHO declared a global pandemic on March 11, 2020) [3–5]. Also, COVID-19 is a new disease born from viral recombination which occurred recently, and that collective memory has forgotten the great epidemics of previous centuries [6, 7]. Several researchers have found that the COVID-19 pandemic has an impact not only on health but also on the environment, economy, education, and human psychology (Figure 1). Acharya et al. (2021) reported that the gradual spread of COVID-19 and insufficient capacity of hospitals has led to the growth of home care which causes a major source of waste contaminated with the virus and subsequent disruption of municipal solid waste management [8]. Donzelli et al. (2021) have shown that, in many cities around the world, the streets are cleared of their cars and passers-by, factories have closed, and many flights have been canceled, implying a significant drop in emissions from toxic gases and consequently improved air quality [9]. Chirani et al. (2021) have shown that the increase in waste that comes from infected people and hospitals leads to the deterioration of water quality which could turn into a source of transmission of the virus [10]. However, the implementation of actions and decisions to control the virus has resulted in the reduction of economic activities following the shutdown of most businesses and consequently the reduced use of public transport and the overall decrease in consumption of electricity, thus implying a decline in the production of thermal and/or nuclear power stations and an increase in renewable energies in the electricity mix [11]. In this review article, we have summarized the influence of COVID-19 on health, as well as some alternative nutrients that can help infected people strengthen their immune systems. On the other hand, we discussed the advantages and disadvantages of COVID-19 in terms of the environment (water, air, waste management, and energy consumption), as well as the influence of the pandemic on the human psyche, the education system, and the global economy (Figure 1). We also have proposed recommendations to mitigate the various adverse consequences of the pandemic. Finally, we have mentioned some bibliographic hypotheses of the influence of climate on the spread of the virus.

## 2. Background Information on Coronavirus Disease

### 2.1. Impact of COVID-19 on Health

The novel coronavirus pandemic is the biggest public health crisis the world has faced in more than a century. Highly contagious and infectious SARS-CoV-2 causes bioaerosols that transport pathogenic microorganisms, thus affecting public health [12]. Clinical symptoms of COVID-19 are respiratory or cardiovascular complications [13]. Bouhanick et al. (2020) assume that infected diabetic patients are more at risk of severe pneumonia with an advanced proinflammatory and prothrombotic state [14]. According to Li et al. (2020), cardiovascular disease is also a risk factor for the progression and prognosis of COVID-19 [15]. The latter, when infected with the disease, may present with severe pneumonia. Indeed, the release of enzymes linked to tissue damage exposes the patient to a greater risk of cytokines by causing a hypercoagulable state [15]. The research team of Segars et al. (2020) describes the state of coronaviruses and their impacts on human reproduction, in particular the behavior of male and female gametes [16]. According to Saqrane and El Mhammedi (2020), SARS-CoV-2, which is a virus belonging to the large coronavirus, is responsible for precise respiratory distress [17].

### 2.2. Nutritional Support for COVID-19 Patients

Good nutrition is central to the regulation of immunity for patients infected with COVID-19. The right choice of foods helps to balance the immune system and optimize its function. In addition, an optimal nutritional diet can positively control oxidative stress. For this reason, it is recommended to choose a predominantly vegetable diet rich in antioxidants, to privilege foods with a low glycemic load, to prefer cooking foods with gentle steam, to favor organic food without contaminants, to practice intermittent fasting, and to take care of the hygiene of life (practice physical activities, avoid the consumption of alcoholic beverages and tobacco, meditate, and think positively).  Table 1 summarizes some of the foods recommended by several researchers and their positive effects on the immune mechanism.

## 3. Impact of COVID-19 on the Environment

### 3.1. Impact of COVID-19 on Water Quality

**Figure 2:**
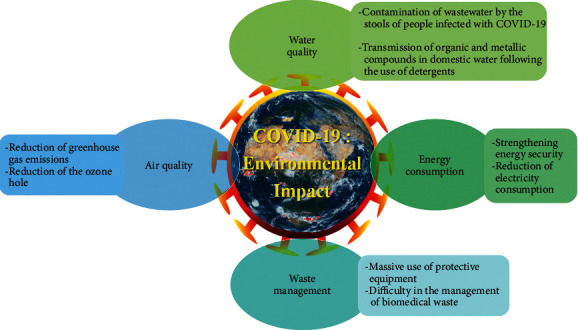
Impact of COVID-19 on the environment.

Population growth and the increase in agroindustrial activities are creating increasing pressure on the planet's freshwater reserves [28, 29]. Indeed, these activities generate a great diversity of pollutants that flow into the water cycle, jeopardizing the fragile natural balance that has allowed life to develop on earth [30]. From an environmental point of view, the aquatic environment is the favorite site for the reception of very complex human and industrial waste [31]. This waste generates more and more pollution, threatening the environment and human health [32, 33]. All countries in the world are concerned with safeguarding freshwater resources, either because they lack water or because they pollute it [34]. The disparity between the needs and the availability of water requires imagining new means of transport and treatment to increase the availability of resources [35]. Protecting water resources has become an even more complicated challenge due to the COVID-19 pandemic which has negatively impacted water quality: First, there is a great possibility of transmitting and detecting ribonucleic acid of SARS-CoV-2 in wastewater through the stools of people infected with this virus [36]. Second, the high consumption of water and the high use of detergents in the period of COVID-19 allowed the transmission of several organic and metallic compounds in domestic waters and consequently the degradation of the water quality [10] (Figure 2). For this reason, decision makers recommended to control new harmful species present in water during the period of COVID-19 and to carry out strategies for the sustainable management of water resources.

### 3.2. Impact of COVID-19 on Air Quality

Despite the harmful effects of COVID-19, it also has positive indirect effects on the environment, including improving air quality by reducing greenhouse gas emissions such as sulfur dioxide, nitrogen oxides, and particulate matter resulting from anthropogenic human activity such as waste incineration and fuel combustion [9, 37, 38]. Several studies suggest that industrial limitations following the COVID-19 health crisis are the main causes of reduction in ambient air pollutants except for ozone [39]. Indeed, the increase in ozone concentration could be linked either to the decrease in ambient nitrogen oxides in urban areas in which volatile organic compounds are limited or to the reported reductions in airborne particles which are responsible for solar activity [40–44]. In addition, during sanitary containment, fewer ambient suspended particles would constitute a less-efficient sink for hydroperoxy radicals, thus increasing the production of ozone-induced by proxy radicals [45–47]. During the short period of confinement, the shutdown of several industries leads to a reduction in the large quantities of atmospheric pollutants resulting from the combustion of carbon, in particular carbon oxides, sulfur oxides, nitrogen oxides, particles in suspension, and heavy metals. Wang and Su (2020) show that nitrogen oxides react with other chemicals to form acid rain [48]. During the lockdown, air quality in all countries of the world has improved remarkably thanks to the strict restriction and adoption of quarantine measures and traffic control (Figure 2).

### 3.3. Impact of COVID-19 on Waste Management

The spread of the health crisis of the COVID-19 pandemic has caused an increase in the use of single-use protective equipment posing massive pressure and significant challenges in the waste management sector [49]. The daily lifestyle and eating habits of the majority of people have undergone a drastic change due to the consumption of food during this pandemic period [50–53]. Furthermore, this epidemic is leading to the emergence of other additional sources of waste which cause complexities in the management of municipal solid waste for governments and organizations that have collected and sorted the waste [54–56]. Frequent use of personal use products and panic shopping is reported to trigger high environmental contamination generated by plastic waste [57]. This latter waste is associated with the need to package requests for the distribution and take-out of food or medical use [58, 59]. Some researchers have found that most people mix COVID-19 protective gear with household waste, which can cause the virus to spread [8, 60]. In addition, during this health crisis, the world has seen a great increase in the amount of biomedical waste generated such as human tissues, body fluids, cotton swabs, bandages, needle syringes, blood bags, and disposable materials (masks, gloves, gowns, hair covers, etc.) [61] (Figure 2). Generally, for good management of solid waste, it would be preferable to recommend (1) carrying out statistical studies on the rate of waste production while covering the different sources of production including hospitals and laboratories [62, 63], (2) separation of the different types of waste at the source to put potentially infected waste in hermetically sealed bags and to recycle uncontaminated waste using safe practices as improper sorting could lead to increased costs of their management [64, 65], and (3) the implementation of special regulations on the statistical data of medical waste collected during the confinement period [66].

### 3.4. Impact of COVID-19 on Energy Consumption

All sectors of industry and transportation were closed during containment, resulting in a significant reduction in energy demand and consumption, enhancing the energy security that has been exploited by the medical industry for manufacturing the products, medical and personal protective equipment [67]. The restriction of mobility and consequently the closure and/or partial operation of transit stations have resulted in the reduction of electricity consumption [68]. In addition, this drop in demand for electricity could also be attributed to the increase in the predominant contribution of renewables in the electricity mix instead of nuclear, coal, and natural gas [69]. Generally, the reduction of industrial activities has decreased energy consumption all over the world and reduced environmental pollution during the period of COVID-19 (Figure 2).

## 4. Impact of COVID-19 on Human Psychology

The rapid spread of the COVID-19 pandemic has led to a high death rate and, therefore, negatively impacts mental health, thus causing social concerns due to government restrictions (confinement, curfew, etc.) [70, 71]. Therefore, the symptoms of distress, depression, posttraumatic stress disorder, anxiety, frustration, and suicide could stem from the length of the duration of the sanitary measures taken to control the virus [72, 73]. To overcome these psychological problems, it is recommended to train psychologists and social workers in the management of the effects of pandemics and health emergencies [74, 75] and to sensitize patients to consult psychologists to reduce the risk of contagion [76, 77].

## 5. Impact of COVID-19 on the Education System

During the COVID-19 period, government officials and policymakers have closed universities and public and private schools to control the spread of the virus by replacing the traditional teaching method with teaching online by maintaining the use of interactive educational tools including platforms for the creation of skills development courses and programs [78, 79]. These tools have a host of benefits that stimulate student learning during this critical time [80]. First of all, these remote educational means allowed us to avoid the White Year and its economic and social repercussions. In addition, these distance courses are more flexible and more suitable for students with physical disabilities as they only require reduced mobility [81]. Finally, the spirit of engagement and self-exploratory learning could gradually develop through this new educational technology [82]. However, distance education pedagogy is not without its drawbacks [83, 84]: First, some low-income schools have not been able to gain access to online education solutions despite efforts and commitments to address the learning loss. Second, the technophobia, the unavailability, and the lack of follow-up and supervision by some parents in this period make learning more complicated in children, especially for those who have difficulty adapting to the new educational environment, and/or their critical economic and social situation does not allow them to dispose of and purchase online learning devices. Third, poor Internet connectivity will hamper communication between teachers and their students. To improve the quality of education, we recommend (1) developing new policies to support the entry of young graduates into the labor market and avoid unemployment, (2) improving the connection speed and the audiovisual quality of the platforms used, (3) prerecording course videos for later use, (4) educating the parents of students about the use of parental controls on technological devices, (5) examining the plagiarism of responses from students and/or candidates assessed remotely, and (6) free provision of electronic and technical equipment and resources for people with limited individual incomes.

## 6. Socioeconomic Impact of COVID-19

The global health crisis of COVID-19 has imposed social isolation where citizens of different countries are prohibited from going out and carrying out their usual activities, thus harming the global economic situation [70]. The consequences of the health restrictions suddenly put in place are the reduction in tourist activity, the weakening of industrial deliverability, the fall in demand from abroad, the dismissal of people, and the reduction of the human budget [85]. To alleviate the economic impact of the pandemic, we recommend (1) building trust among citizens by authorities by communicating honestly, (2) improving the quality and access to essential services through the development of digital payments to reach vulnerable populations who work in the informal economy or do not have a bank account, (3) protecting businesses and families from the risk of eviction and bankruptcy, (4) the search for long-term social, economic, and environmental cobenefits as part of their stimulus investments, (5) the creation of jobs for the benefit of the unemployed and young graduates, (6) taking into account the capacity of a project to directly replace failing demand and its impact on import levels or the country's trade balance, (7) the organization of interventions to strengthen the capacities of societies and economies to face an external shock and to overcome it like the current COVID-19 pandemic and also other forms of disasters' natural factors and the future effects of climate change, (8) support and generalization of green technologies by investing in networks that facilitate the use of renewable energies and electric vehicles or low-tech solutions, such as reforestation or restoration and management of landscapes and watersheds without incurring significant costs for the economy in the decades to come due to the depreciation of assets, and (9) supporting politicians on the road to recovery.

## 7. Impact of Climate on the Spread of COVID-19

Several studies confirm the effects of air temperature and humidity on the coronavirus [86–88]. The researchers were able to establish the existence of a causal link between the climatic conditions and the number of new positive cases and deaths. A study by Mesay Moges Menebo (2020) in Oslo (capital of Norway) states that temperature and precipitation are correlated with the incidence rate of daily cases of COVID-19 at maximum and normal temperatures and positively associated with COVID-19 while precipitation is negatively associated [89]. According to K. H. Chan et al. (2011), coronaviruses do not survive in high-temperature countries such as Malaysia, Indonesia, and Thailand, while the spread is intensive in low-temperature countries [90].

## 8. Conclusions

In response to the COVID-19 pandemic, government officials and policymakers have compulsorily implemented lockdown measures that have influenced the environmental and economic situation, as well as human psychology and the educational education system in the whole world. From an environmental perspective, reductions in transport and mobility have reduced greenhouse gas emissions and reduced demand for industrial and commercial energy. In addition, the poor management of waste and the decrease in water quality in this period of COVID-19 are due to the lack of awareness of citizens. The health restrictions suddenly put in place lead to the deterioration of human psychology, the modification of the education system, the reduction of tourist activity, the dismissal of employees, and the decrease in the human budget and the gross domestic product.

## Figures and Tables

**Table 1:** Nutritional support for COVID-19 patients.

Type of nutrition or diet	Food description	Reference
Fish oil	Lipid emulsions: a large amount of energy in a small volume; high proportions of omega-3 acids are precursors to anti-inflammatory mediators such as eicosapentaenoic and docosahexaenoic acids	[18, 19]

Vitamin C	Vitamin C could have a double effect: antioxidant: protects cells and tissues in the body from oxidative damage and dysfunction; immunoprotective: inhibits the secretion of lactate produced by activated immune cells and protects the innate immunity from angiotensin 2	[20, 21]

Vitamin D	Vitamin D improves lung function in patients with asthma, chronic obstructive pulmonary disease, or in smokers, especially if there is a vitamin D deficiency at the start. Calcidiol (calcidiol, 25-hydroxycholecalciferol, or 25-hydroxyvitamin D) has reliable intestinal absorption (close to 100%) and can quickly restore serum concentrations because it does not require hepatic 25-hydroxylation. When calcitriol (a hormonal metabolite of vitamin D) enters the nuclear receptor, a deoxyribonucleic acid-binding protein interacts with regulatory sequences near target genes and recruits active chromatin complexes that genetically and epigenetically alter production transcriptionally. Calcitriol regulates serum calcium concentration and, therefore, feeds back with parathyroid hormone. Vitamin D provides a physical barrier, natural cellular immunity through the induction of antimicrobial peptides, and a modulator of adaptive immunity against colds. Vitamin D reduces the production or expression of proinflammatory cytokines and increases the expression of anti-inflammatory cytokines by macrophages	[22–24]

Zinc	Improves immune function, improves resistance to infections, and increases the cytotoxic activity of natural killer cells, which can attack cells that have abnormal or unusual proteins in the plasma membrane. It is an anti-inflammatory agent, maintaining immune tolerance because it induces the development of Treg cells and attenuates the development of proinflammatory helper T cells 17 and helper T cells 9, in addition to being involved in the production of antibodies, in particular immunoglobulins *G*	[23, 25]

Vitamin A	Antiinfective vitamin	[26]

Required water	Maintaining adequate hydration remotes heart or renal failure and recent clinical history as diarrhea, vomiting, and electrolyte imbalances	

Probiotics	Probiotics may be useful before intestinal dysbiosis with the decrease in bifidobacteria and lactobacilli. Micronutrient support of the intestine as well as the administration of a symbiotic (probiotics and prebiotics) restore balance and prevent the risk of secondary infection. In chronic inflammatory diseases, taking a high-concentration probiotic reduces the plasma levels of proinflammatory cytokines at the expense of those regulating inflammation, with changes in the fecal microbiota	[27]

## Additional Points

This manuscript describes an original work with the aim to considerable efforts being deployed to develop medicines to treat and vaccines to prevent the disease as well as the recommendations made by some governments around the world. Among these countries, those applied by Morocco have proven their effectiveness and made the impact of the epidemic much less significant than in other neighboring countries.
